# Supplementary figures and images for: Enhancing phishing detection with dynamic optimization and character-level deep learning in cloud environments
Source: PeerJ Comput Sci. 2025 May 19;11:e2640. doi: 10.7717/peerj-cs.2640 (PMC12190431; doi:10.7717/peerj-cs.2640)

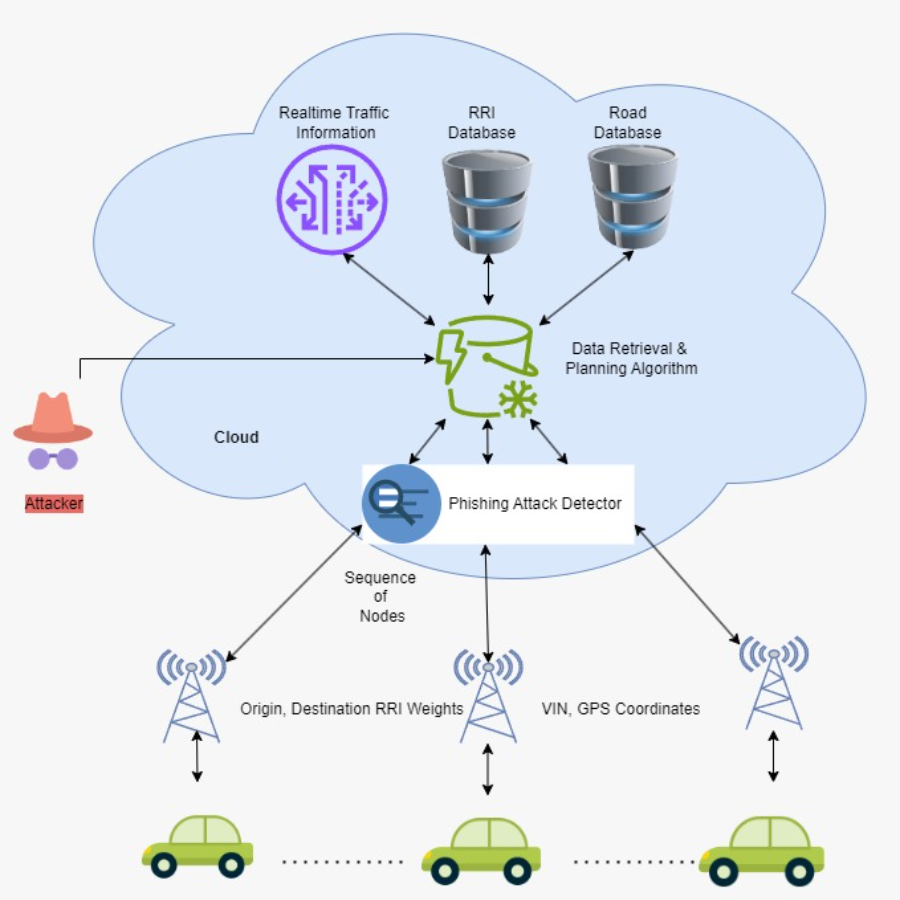

Supplement: Supplemental Information 1 [file peerj-cs-11-2640-s001.png]

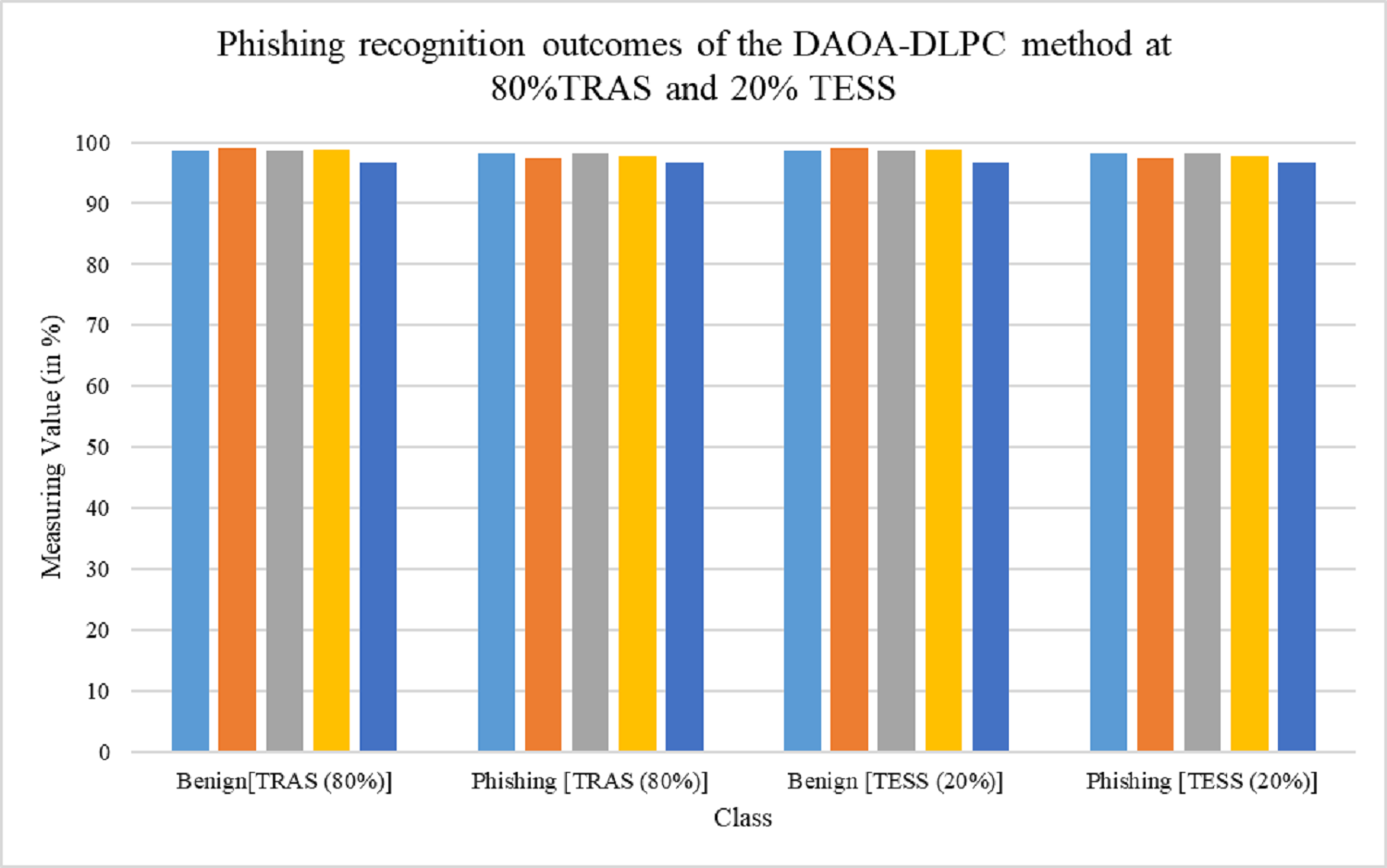

Supplement: Supplemental Information 2 [file peerj-cs-11-2640-s002.png]

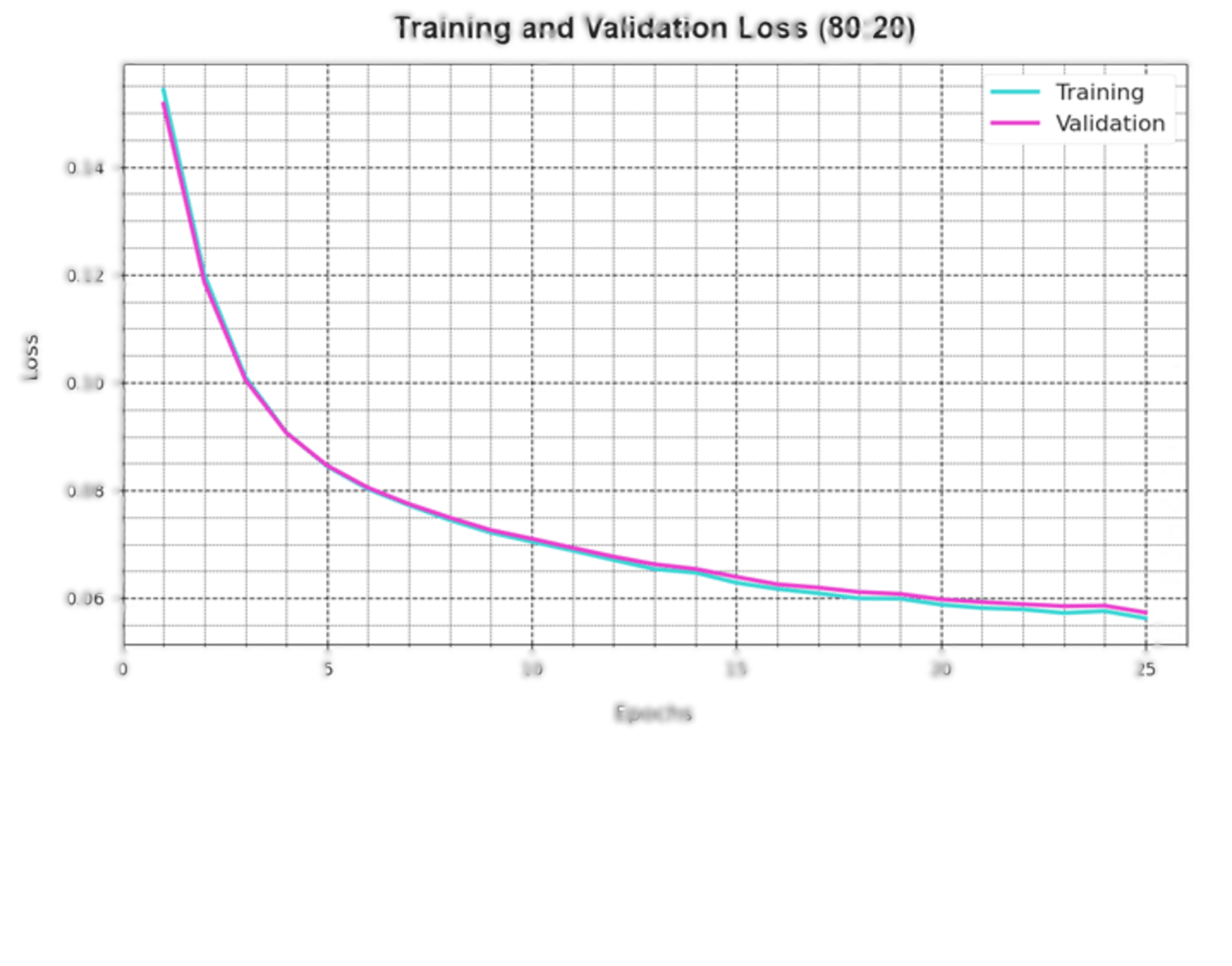

Supplement: Supplemental Information 3 [file peerj-cs-11-2640-s003.png]

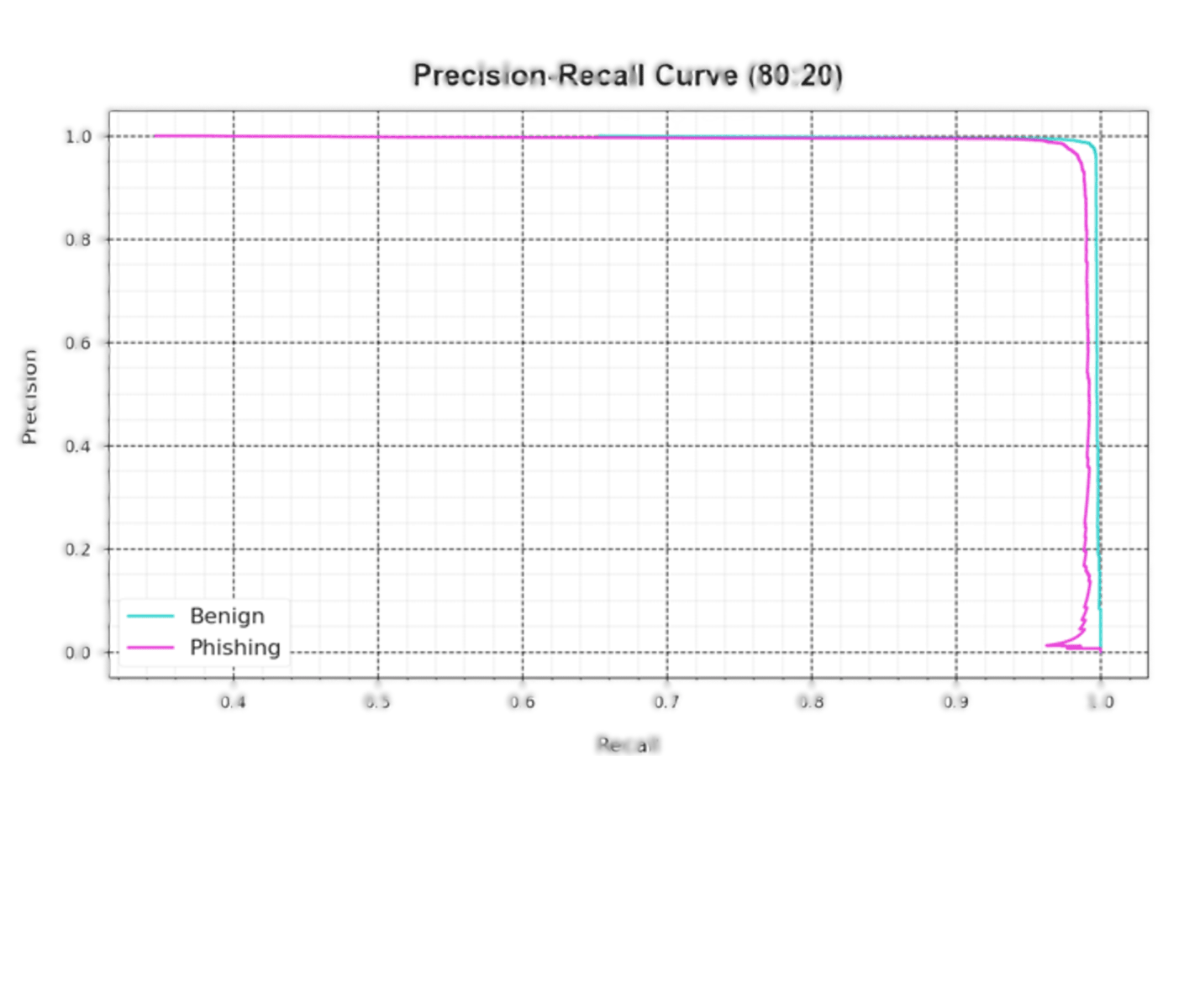

Supplement: Supplemental Information 4 [file peerj-cs-11-2640-s004.png]

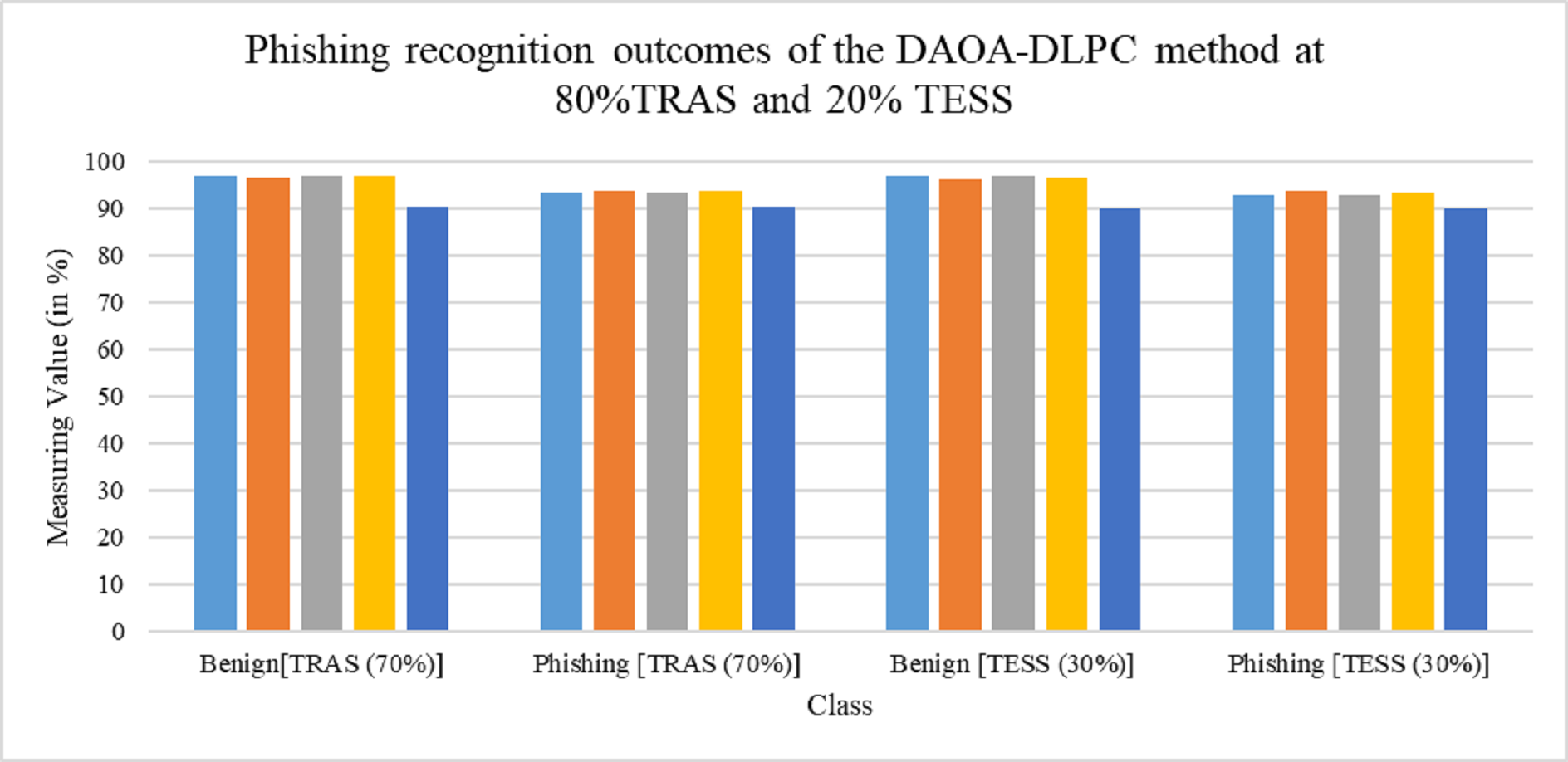

Supplement: Supplemental Information 5 [file peerj-cs-11-2640-s005.png]

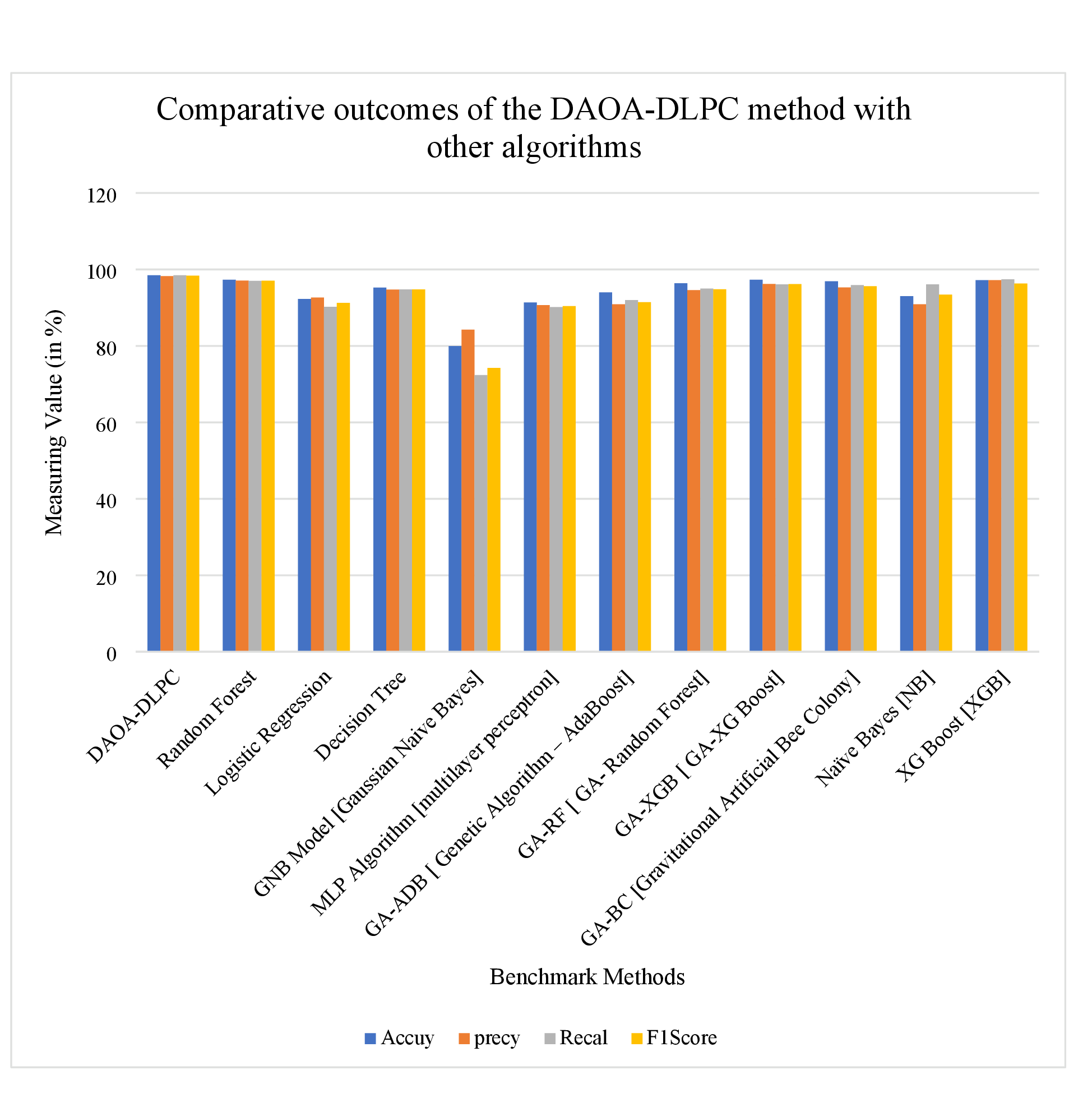

Supplement: Supplemental Information 6 [file peerj-cs-11-2640-s006.png]
