# Supplementary material for: Enhancing phishing detection with dynamic optimization and character-level deep learning in cloud environments
Source: PeerJ Comput Sci. 2025 May 19;11:e2640. doi: 10.7717/peerj-cs.2640 (PMC12190431; doi:10.7717/peerj-cs.2640)
Supplement: Supplemental Information 7 [file peerj-cs-11-2640-s007.docx]

**Table 2.** Details of the dataset.

| Class | No. of Instances |
| --- | --- |
| Benign | 58000 |
| Phishing | 30646 |
| **Total** | **88646** |
